# Probing hippocampal stimulation in experimental temporal lobe epilepsy with functional MRI

**DOI:** 10.3389/fnimg.2024.1423770

**Published:** 2024-08-14

**Authors:** Niels Schwaderlapp, Enya Paschen, Pierre LeVan, Dominik von Elverfeldt, Carola A. Haas

**Affiliations:** ^1^Division of Medical Physics, Department of Diagnostic and Interventional Radiology, Faculty of Medicine, University Medical Center Freiburg, University of Freiburg, Freiburg im Breisgau, Germany; ^2^BrainLinks-BrainTools Center, University of Freiburg, Freiburg im Breisgau, Germany; ^3^Experimental Epilepsy Research, Department of Neurosurgery, Faculty of Medicine, Medical Center - University of Freiburg, University of Freiburg, Freiburg im Breisgau, Germany; ^4^Department of Radiology and Paediatrics, Cumming School of Medicine, Hotchkiss Brain Institute, University of Calgary, Calgary, AB, Canada

**Keywords:** DBS, fMRI, epilepsy, kainate mouse model, TLE, hippocampus

## Abstract

Electrical neurostimulation is currently used to manage epilepsy, but the most effective approach for minimizing seizure occurrence is uncertain. While functional MRI (fMRI) can reveal which brain areas are affected by stimulation, simultaneous deep brain stimulation (DBS)-fMRI examinations in patients are rare and the possibility to investigate multiple stimulation protocols is limited. In this study, we utilized the intrahippocampal kainate mouse model of mesial temporal lobe epilepsy (mTLE) to systematically examine the brain-wide responses to electrical stimulation using fMRI. We compared fMRI responses of saline-injected controls and epileptic mice during stimulation in the septal hippocampus (HC) at 10 Hz and demonstrated the effects of different stimulation amplitudes (80–230 μA) and frequencies (1–100 Hz) in epileptic mice. Motivated by recent studies exploring 1 Hz stimulation to prevent epileptic seizures, we furthermore investigated the effect of prolonged 1 Hz stimulation with fMRI. Compared to sham controls, epileptic mice showed less propagation to the contralateral HC, but significantly stronger responses in the ipsilateral HC and a wider spread to the entorhinal cortex and septal region. Varying the stimulation amplitude had little effect on the resulting activation patterns, whereas the stimulation frequency represented the key parameter and determined whether the induced activation remained local or spread from the hippocampal formation into cortical areas. Prolonged stimulation of epileptic mice at 1 Hz caused a slight reduction in local excitability. In this way, our study contributes to a better understanding of these stimulation paradigms.

## 1 Introduction

Deep brain stimulation (DBS) is increasingly used for the treatment of intractable epilepsy (Fisher and Velasco, 2014; Foutz and Wong, 2022; Velasco et al., 2022). Mesial temporal lobe epilepsy (mTLE) in particular, the most common form of focal epilepsy in adults, is often refractory to anti-seizure medication. In these cases, surgical resection of the seizure focus is often performed to attain seizure freedom, but there is no guarantee that surgery will result in a positive long-term outcome (Wiebe et al., 2001; Immonen et al., 2010; Hemb et al., 2013; Mohan et al., 2018). DBS may represent an alternative treatment and can be beneficial in epilepsy via two mechanisms: First, high-frequency stimulation (HFS) initiated at the onset of an epileptic seizure could desynchronize neuronal activity and thus terminate the seizure or at least prevent further propagation (Yu et al., 2018). Second, low-frequency stimulation (LFS) could lead to a reduction of neuronal excitability and thus reduce the probability of seizure occurrence (Lim et al., 2016; Paschen et al., 2020; Koubeissi et al., 2022). While there is promising evidence that stimulation has a positive therapeutic effect, it does not always achieve complete seizure freedom (Boon et al., 2007; Klinger and Mittal, 2018; Li and Cook, 2018). A current challenge is to better investigate the effects of stimulation in order to increase effectiveness and minimize side effects.

The outcome of stimulation is largely influenced by the stimulation site and the exact stimulation parameters. In mTLE, the hippocampus (HC) is frequently identified as the presumed site of seizure onset in many patients, making it a prominent target for intervention (Tellez-Zenteno et al., 2006; Velasco et al., 2007; Cukiert et al., 2014, 2017; Lim et al., 2016). Initial stimulation parameters are selected based on experience. If the response is sub-optimal or side effects occur, parameters such as amplitude or frequency are adjusted to individual patients on a trial-and-error basis (Simpson et al., 2022). This time-consuming process is complicated by the fact that it is usually unknown which brain regions and networks are affected by the stimulation. In addition, it is difficult to identify and adapt to long-term changes over time (Kokkinos et al., 2019; Khambhati et al., 2021).

A valuable method for directly assessing the brain-wide responses to stimulation is functional MRI (fMRI), which has been performed concurrently with DBS in recent studies (Jones et al., 2014; Thompson et al., 2020; Middlebrooks et al., 2021; Loh et al., 2022). However, due to the difficulty of performing DBS and fMRI simultaneously, published reports from epilepsy patients are scarce. For example, stimulation in the anterior nucleus of the thalamus showed a more widespread activation at 145 Hz than at 30 Hz stimulation (Middlebrooks et al., 2021). In another study, similar activation patterns were observed with high (8 mA) and low (4 mA) currents at 20 Hz stimulation, whereas no significant fMRI response was found in patients with 1 Hz stimulation (Jones et al., 2014). Further studies are needed to better understand the influence of stimulation parameters.

This investigation can be accelerated by preclinical DBS-fMRI studies. Studies targeting the perforant path or the HC showed fMRI activations within the hippocampal structure and in additional areas, e.g., thalamus and septum, at stimulation frequencies of 10–130 Hz, but no fMRI response at 2 Hz electrical stimulation (Angenstein et al., 2007; Canals et al., 2008; Berge et al., 2015). However, these studies were performed in healthy rodents. Since severe structural alterations in the sclerotic HC have been found in rodent models of mTLE (Häussler et al., 2016; Janz et al., 2017a) and human mTLE (Thom et al., 2002; Freiman et al., 2021), it remains unclear to what extent these results, in particular the frequency dependency and the spread of activation from the HC to connected areas, reflect the situation in mTLE.

The alterations of the HC that occur in the human disease are replicated particularly well in the intrahippocampal kainate (ihKA) mouse model of mTLE, including the onset of spontaneous recurrent seizures and robust unilateral hippocampal sclerosis following status epilepticus (Bouilleret et al., 1999; Riban et al., 2002; Häussler et al., 2012). Hippocampal sclerosis in mTLE patients is characterized by neuronal cell loss and reactive gliosis and is often associated with granule cell dispersion and mossy fiber sprouting (Blümcke et al., 2013; Thom, 2014). Similarly, ihKA-treated mice show an extensively loss of pyramidal neurons in CA1/CA3 regions and interneurons throughout the sclerotic hippocampus, and preservation of dentate granule cells with their entorhinal inputs, and CA2 pyramidal cells (Häussler et al., 2012, 2016; Marx et al., 2013; Janz et al., 2018). As in mTLE patients, the sclerotic hippocampus is thought to be the focus of epileptic activity in ihKA mice (Pallud et al., 2011; Krook-Magnuson et al., 2013, 2015). Results obtained from the ihKA model of mTLE can therefore be translated well to human mTLE (Lévesque and Avoli, 2013). However, to our knowledge, hippocampal stimulation in this model has not been investigated with fMRI.

Non-imaging studies in experimental TLE examined the impact of electrical stimulation in the HC on the rate of epileptiform activity. Long-term (several days) HFS was found to be most effective in reducing seizure rates (Bragin et al., 2002; Wyckhuys et al., 2010; Van Nieuwenhuyse et al., 2015), but also LFS (1–5 Hz) showed promising results (Rajdev et al., 2011; Rashid et al., 2012; Salam et al., 2016; Paschen et al., 2020).

In a recent study, we found that hippocampal LFS in the ihKA model of mTLE reduces epileptic seizures without affecting behavior or memory performance (Paschen et al., 2023). Motivated by this, we used fMRI and EEG in this study to further investigate electrical stimulation in the ihKA model of mTLE.

In this study, we show (1) which regions respond to electrical HC stimulation in chronically epileptic and sham control mice, (2) the influence of different stimulation parameters on the brain-wide activation pattern, and (3) the changes in fMRI responses induced by prolonged 1 Hz stimulation.

## 2 Materials and methods

### 2.1 Animals

All experiments were performed with adult (18–24 weeks) male mice (*n* = 12) that were negative littermates of transgenic mouse lines (C57BL/6 background) according to the 3R principle. All animal procedures were carried out under the guidelines of the European Community's Council Directive (2010/63/EU) and were approved by the regional council (Regierungspräsidium Freiburg, G-19/04).

### 2.2 Kainate injections

Mice were injected with 50 nl kainate (Tocris, Bristol, UK) (*n* = 6) or saline (*n* = 6) into the right dorsal HC. In brief, the stereotaxic injection was performed under deep anesthesia (ketamine hydrochloride 100 mg/kg, xylazine 5 mg/kg, atropine 0.1 mg/kg body weight, i.p.) using Nanoject III [Drummond Scientific Company, Broomall, PA, USA, as described previously (Paschen et al., 2020)]. Mice were randomly assigned for kainate (15 mM in 0.9% sterile saline) or saline injection. Stereotaxic coordinates relative to Bregma (in mm) were: anterioposterior (AP) = −2.0, mediolateral (ML) = −1.5, and relative to the cortical surface: dorsoventral (DV) = −1.5. Following kainate injection the occurrence of a behavioral status epilepticus was verified, characterized by mild convulsion, chewing, immobility, or rotations, as described before (Riban et al., 2002; Tulke et al., 2019).

### 2.3 Construction of electrodes

The stimulation/recording electrodes were specifically designed according to Duffy et al. (2015) and Dunn et al. (2009) and made of carbon to minimize susceptibility artifacts during MRI measurements. To this end, sections of 1k carbon fiber tow (CST Composites, Tehachapi, California, USA), consisting of 1,000 carbon filaments, were split five times to achieve bundles of ~0.2k. For the electrodes serving as ground/reference, four of these 1k tows were put together to achieve a lower current density and to prevent stimulation at the ground/reference electrodes. The carbon bundles were cold-soldered to sections of copper wire using conductive silver epoxy (MG Chemicals, Ontario, Canada) and coated with two layers of polydimethylsiloxane (PDMS, Sylgard 184, The Dow Chemical Company, USA) to ensure a biocompatible insulation. The carbon ends were cut to 3 mm length to expose the contacts prior to implantation.

### 2.4 Electrode implantations

Sixteen days after kainate/saline injections, electrodes were implanted into the ipsilateral and contralateral dorsal HC (HCi and HCc, respectively). Stereotaxic coordinates are given relative to Bregma in mm for anterioposterior (AP) and mediolateral (ML) or to the cortical surface for dorsoventral (DV) coordinates: HCc: AP = −2.0, ML = +1.4, DV = −1.6; HCi: AP = −2.0, ML = −1.4, DV = −1.6. Two thick carbon fiber bundles were placed above the frontal cortex to provide reference and ground. The implants were fixed with dental cement (Paladur, Kulzer GmbH, Hanau, Germany).

### 2.5 MRI acquisition

MRI experiments were performed approximately 2 weeks after electrode implantation. Anesthesia was initiated with ca. 5 % sevoflurane (AbbVie, Ludwigshafen, Germany) and maintained at ca. 2 % during setup. Subsequently, the anesthesia was switched to medetomidine (Domitor, Vetoquinol, Germany; s.c. bolus 0.3 mg/kg, followed by continuous s.c. infusion 0.6 mg/kg/h) (Adamczak et al., 2010; Niranjan et al., 2016). Animals breathed freely during the scans (30 % O_2_, ca.120–170 breaths/min). A water heater connected to tubes in the animal bed was used to maintain the body temperature at 36°C (maximum deviations of ± 1.5°C were observed). After the scans, atipamezole (Antisedan, Vetoquinol, Germany) was administered (s.c., 0.8 mg/kg) to reverse the sedative effect of medetomidine.

This type of anesthesia was chosen to minimize the influence of anesthesia on the fMRI results. A common alternative would be the combination of isoflurane and medetomidine (Grandjean et al., 2014; Pradier et al., 2021). The disadvantage of this anesthesia for our study would be that isoflurane prevents epileptic activity (Airaksinen et al., 2010) and reduces the BOLD amplitude in the case of spontaneous breathing (Alst et al., 2019). The advantage of medetomidine alone is that it does not suppress epileptic activity (Airaksinen et al., 2012), however, due to the low level of sedation, animals are more likely to move during scanning. In this study, strong movements during the scan could be identified by the signal trace of the pressure sensor (which was used to detect breathing) and by artifacts in the EEG. Each fMRI experiments lasted ~6 min (see Section “2.8 Electrical Stimulations”). If strong movements occurred during this time, the scan was stopped and restarted when the animal came to rest again. If the movements continued, the session was ended and repeated on another day. In total, the individual sessions lasted approximately 1 h (including adjustments, reference scans and two to four fMRI scans).

MRI data were acquired on a preclinical 7 T system (BioSpec 70/20, Bruker BioSpin, Ettlingen, Germany) using a 2-channel surface transmit-receive cryoprobe. Adjustments included local shimming up to 3rd order, adapted to the brain volume. An anatomical scan was acquired using a fast spin-echo sequence (RARE) with the parameters: TR 4 s, effective TE 40 ms, RARE factor 4, encoding matrix 120 x 64, in-plane resolution 0.14 × 0.14 mm^2^, number of slices 12, slice thickness 0.8 mm, slice gap 0.2 mm.

FMRI data were acquired using a gradient, multi-echo EPI sequence with the parameters: TR 1.5 s, TE_1_ 14 ms, TE_2_ 23 ms and TE_3_ 32 ms, encoding matrix 60 x 32, in-plane resolution 0.28 x 0.28 mm^2^, number of slices 12, slice thickness 0.8 mm, slice gap 0.2 mm, excitation pulse flip-angle 70°, acquisition bandwidth 250 kHz.

### 2.6 fMRI postprocessing

Data analysis was performed using FSL (FMRIB, Oxford, UK) and Matlab (Mathworks, USA). Preprocessing included motion correction, slice timing correction and high pass temporal filtering (0.01 Hz). The three individual time series (one for each echo) were combined into a single “multi-echo” dataset by T2^*^-weighted combination (Poser et al., 2006) (Supplementary Figure 1). After manual brain extraction, the images were spatially smoothed using a Gaussian kernel with a full-width at half-maximum of 1.5 times in-plane resolution.

The activation was analyzed using the general linear model (GLM) in FSL (Woolrich et al., 2001, 2004). In the first level, the stimulation timings were convolved with finite impulse response functions (8th order, 24 s window) to create the design matrix (Liu et al., 2017). In the group level GLM, a fixed-effects model was used and the results were registered to the AMBMC atlas space (Richards et al., 2011; Janke and Ullmann, 2015).

BOLD fMRI time courses were extracted from several region-of-interests of the AMBMC atlas. First, the time series of all voxels in the respective atlas regions were averaged. Then the individual stimulations were averaged, whereby any offsets before the start of the stimulations were removed.

### 2.7 Local field potential recordings

During fMRI measurements, local field potential (LFP) signals were recorded (EGI, Electrical Geodesics, USA) in the left dorsal HC with a sampling rate of 1 kHz. The data were analyzed with the toolbox EEGLAB (Delorme and Makeig, 2004) in Matlab (Mathworks, USA). Post-processing included removal of the 50 Hz line noise, a moving average FIR filter (order 20) to remove residual line noise and MRI gradient artifacts, and temporal high pass filtering at 2 Hz.

### 2.8 Electrical stimulations

The stimulation (STG1004, Smart Ephys/Multi-Channel Systems, Reutlingen, Germany) was performed through the electrode in the right dorsal HC with charged balanced bi-phasic pulses; 400 μs phase duration, anodic first.

A single block-design experiment consisted of four 10 s stimulation blocks and 60 s rest periods, except for the 1 Hz stimulation, which lasted 20 s. The scan time of these experiments were 5 min 40 s and 6 min 20 s, respectively.

For systematic parameter testing, amplitude effects were first investigated by keeping a constant stimulation frequency of 10 Hz while increasing the amplitude in 50 μA increments from 80 to 230 μA over several experiments. Based on these experiments, the amplitude was then kept constant at 130 μA and the frequency was set to 1, 40 and 100 Hz.

Finally, it was tested whether a prolonged 1 Hz stimulation causes a sustained effect. For this purpose, continuous 1 Hz stimulation at 130 μA for 10 min was performed inside the MRI system but without running fMRI. Block-design experiments with 10 Hz at 130 μA, before and after the 1 Hz stimulation were used as readout.

### 2.9 Transcardial perfusion and immunohistochemistry

At the end of the experiments, mice were deeply anesthetized and transcardially perfused with 4% paraformaldehyde in 0.1 M phosphate buffer. Following dissection, brains were sectioned on a vibratome and processed for immunohistochemistry to verify hippocampal sclerosis and electrode positions as described (Paschen et al., 2020).

### 2.10 Statistical analysis

Statistical analysis was performed using FSL (v5, FMRIB, Oxford, UK) and Matlab [Version 9.1.0 (R2016b), The MathWorks Inc., Natick, MA, USA]. All fMRI activations were displayed using FSL's Gaussian Random Field (GRF)-theory-based voxel-wise correction at p ≤ 0.05, as recommended in Eklund et al. (2016) and Woo et al. (2014). In order to test for group differences, all subjects in these groups were included in the design matrix (for more details, we refer to the user guide of the FSL function “FEAT”, section “group statistics”). Two control mice had to be excluded because the ground/reference electrodes were not connected to the brain. One kainate mouse died during the period of the experiments. All statistical tests and animal numbers are given in the results and figure captions.

## 3 Results

**Figure 1:**
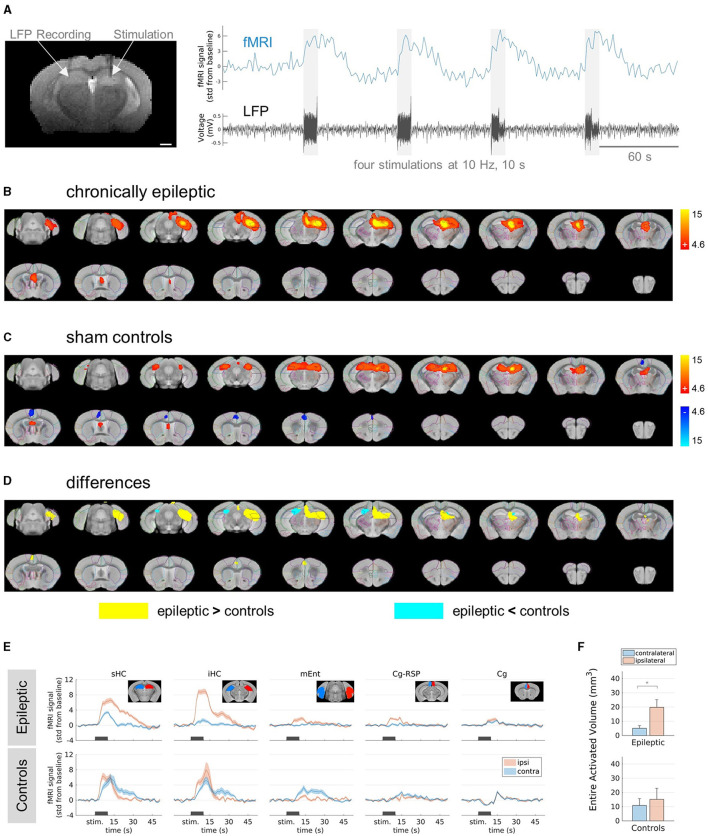
FMRI responses in epileptic mice and sham controls stimulated at 10 Hz. **(A)** Experimental design. **(Left)** Representative T2-weighted anatomical reference image showing stimulation and recording electrodes implanted in the right and left hippocampus, respectively. Scale bar: 1 mm. **(Right)** Exemplary (unprocessed) fMRI time course (blue) showing the responses to the stimulation indicated by the electrical artifacts in the LFP trace (black). Scale bar: 60 s. **(B)** fMRI activation in chronically epileptic mice from caudal (top left) to rostral (bottom right) direction at stimulation parameters: 10 Hz, 10 s, 80 μA. The group-level (*n* = 6) fMRI activations are shown as red-yellow overlays in the AMBMC reference space (gray background) with contours of the AMBMC mouse brain atlas (colors red to yellow represent z-scores from 4.6 to 15 of significant responses at a voxel-wise corrected threshold *p* < 0.05). **(C)** fMRI activation in sham controls stimulated at 10 Hz, for 10 s and 80 μA. Positive and negative responses are shown as red-yellow (z-scores from 4.6 to 15) and blue-light blue (z-scores from −4.6 to −15) overlays (group-level *n* = 4, voxel-wise corrected threshold *p* < 0.05). **(D)** Group-level differences between epileptic (*n* = 6) and control (*n* = 4) mice. Yellow and cyan colored areas represent areas with significantly stronger responses in epileptic mice or controls, respectively (two-sample unpaired *T*-test, voxel-wise corrected threshold *p* < 0.05, stimulation parameters 10 Hz, for 10 s and 80 μA). **(E)** Mean fMRI responses in selected regions (mean ± SEM). The black bars indicate the stimulation periods. Atlas regions: sHC, septal hippocampus; iHC, intermediate hippocampus; mEnt, medial entorhinal cortex; Cg-Rsp, cingulate region/retrosplenial area; Cg, cingulate region/anterior cingulate area. **(F)** Quantification of the entire activated volume on the ipsilateral and contralateral hemisphere (mean ± SEM). In epileptic mice, the activated volumes on the ipsilateral hemisphere were significantly larger than on the contralateral side, whereas no difference was found in sham controls (^*^p < 0.05, one-sample *t*-tests between ipsi- and contralateral “Entire Activated Volumes”).

The stimulations were applied in the septal pole of the ipsilateral HC. Based on our previous study (Janz et al., 2017b), hippocampal sclerosis on this side was identified in kainate-injected mice by a slight increase in signal on the T2-weighted reference images (Figure 1A) and an enlarged dentate gyrus due to granule cell dispersion (Supplementary Figure 2).

### 3.1 Differences between epileptic mice and sham controls in fMRI responses to electrical HC stimulation

First, we delineated the brain regions responsive to electrical HC stimulation in chronically epileptic mice compared to sham controls. We selected a stimulation frequency of 10 Hz. Preliminary tests in an epileptic mouse showed only minimal stimulation artifacts in the LFP and almost no fMRI response at a stimulation amplitude of 30 μA (Supplementary Figure 3). Stimulations at 80 μA elicited robust fMRI activations in epileptic mice (Figure 1B). The main activation was induced around the stimulation site in the ipsilateral septal HC. It was restricted to the ipsilateral dorsal to intermediate HC and did not propagate into the ventral part of the HC nor to the contralateral side. In caudal direction, activation affected parts of the ipsilateral entorhinal cortex and in rostral direction, parts of the cingulate cortex and the septal area.

In controls, no fMRI response was detected at 10 Hz and 30 μA (Supplementary Figures 4A–D). However, the first stimulation block at 80 μA triggered epileptiform afterdischarges (ADs) in control animals (Supplementary Figures 4E, F). The subsequent stimulations did not elicit further ADs (Supplementary Figure 4G). These experiments showed fMRI activation bilaterally in the septal HC, in parts of the entorhinal cortex, septum and a negative BOLD response in the cingulate cortex (Figure 1C).

Testing for group differences revealed areas where epileptic mice showed either significantly stronger or weaker responses compared to sham controls (Figure 1D). The fMRI response in epileptic mice was significantly stronger in the ipsilateral HC and in parts of the ipsilateral thalamus, entorhinal cortex and cingulate cortex (Figure 1D, yellow areas). However, in the contralateral septal HC and in a very small part of the ipsilateral septal HC, the fMRI response was weaker in epileptic mice compared to sham controls (Figure 1D, cyan areas).

The time courses of the BOLD amplitudes further explain the found differences (Figure 1E). While control animals showed similar BOLD responses in the ipsi- and contralateral septal and intermediate HC, epileptic mice showed extremely strong BOLD responses in the ipsilateral HC und only weak responses on the contralateral side. Comparing the entire activated volumes in epileptic mice showed significantly more activation in the ipsilateral hemisphere, whereas sham controls recruited similar activation in both hemispheres (Figure 1F).

In conclusion, the induced fMRI activations differed between sham controls and chronically epileptic mice, with the latter showing pronounced BOLD activity in the ipsilateral hippocampal network and only little spread to the contralateral side. Note that at 10 Hz stimulation, epileptiform ADs occurred only in sham controls and not in epileptic mice.

### 3.2 Influence of the stimulation amplitude on fMRI activation in epileptic mice

**Figure 2:**
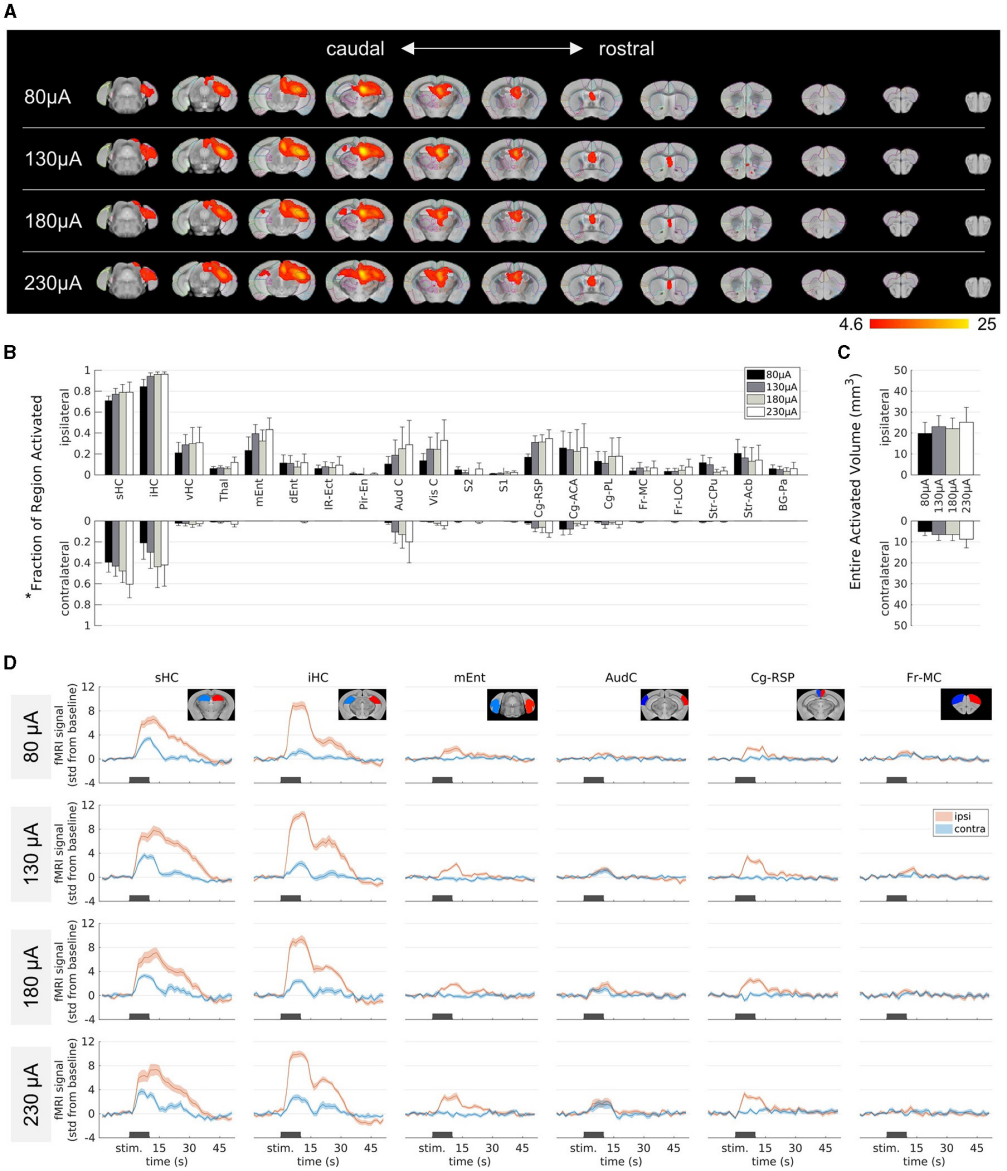
Influence of the stimulation amplitude on fMRI activation in epileptic mice. **(A)** fMRI activation maps for stimulation amplitudes of 80 μA (top row) to 230 μA (bottom row) from caudal **(left)** to rostral **(right)** direction. The group-level fMRI activations are shown as red-yellow overlays in the AMBMC reference space (gray background) with contours of the AMBMC mouse brain atlas (colors red to yellow represent z-scores from 4.6 to 25 of significant responses at a voxel-wise corrected threshold *p* < 0.05; group n = 6 for 80 and 130 μA, n = 4 for 180 and 230 μA). **(B)** Quantification of the activated fractions in atlas regions* for stimulation amplitudes from 80 to 230 μA (mean ± standard error of the mean, SEM). Increasing the amplitude did not result in significant differences of the activated volumes (one-way ANOVA *p* > 0.05 with Tukey-Kramer multiple comparison tests). **(C)** Quantification of the entire activated volume on the ipsilateral and contralateral hemisphere (mean ± SEM). No significant differences were found between the stimulation amplitudes (one-way ANOVA *p* > 0.05 with Tukey-Kramer multiple comparison tests). However, the activated volumes on the ipsilateral hemisphere were significantly larger than on the contralateral side (one-sample t-tests between ipsi- and contralateral “Entire Activated Volumes”; Bonferroni-Holm corrected *p* = 0.016, 0.016, 0.019 and 0.024 for 80, 130, 180 and 230 μA, respectively). **(D)** Mean fMRI responses in selected regions (mean ± SEM). The black bars indicate the stimulation periods. *Atlas regions: sHC, septal hippocampus; iHC, intermediate hippocampus; vHC, ventral hippocampus; Thal, thalamus; mEnt, medial entorhinal cortex; dEnt, dorsal entorhinal cortex; IR-Ect, insular region/ectorhinal area; Pir-En, piriform cortex/endopiriform claustrum; Aud C, auditory/temporal region; Vis C, visual cortex; S2, secondary somatosensory cortex; S1, primary somatosensory cortex; Cg-Rsp, cingulate region/retrosplenial area; Cg-ACA, cingulate region/anterior cingulate area; Cg-PL, cingulate region/prelimbic area; Fr-MC, frontal association/motor cortex; Fr-LOC, frontal/lateral orbital cortex; Str-CPu, striatum/caudate putamen; Str-Acb, striatum/accumbens; BG-Pa, basal ganglia/pallidum.

To analyze the impact of different stimulation parameters on the brain-wide activation pattern in chronically epileptic mice, we kept the stimulation frequency constant at 10 Hz and varied the current amplitudes ranging from 80 to 230 μA during fMRI experiments (Figure 2). FMRI activation was found in the ipsilateral dorsal to intermediate HC, parts of the entorhinal, cingulate cortex and septal area. Increasing the amplitude stepwise from 80 to 230 μA resulted in similar activation patterns with only a slight increase in spread (Figure 2A).

To quantify the extent of activation, the percentage of the area covered by the fMRI activation was calculated in each brain region (Figure 2B). Group comparisons across the different brain regions revealed no significant differences between the different amplitudes (one-way ANOVA *p* > 0.05 with Tukey-Kramer multiple comparison test). The entire activated volume did also not change significantly with amplitude (Figure 2C). A comparison between the two hemispheres revealed significantly larger activation volume on the ipsilateral side at all current amplitudes (one-sample *t*-tests between ipsi- and contralateral “Entire Activated Volumes”; Bonferroni-Holm corrected *p* = 0.016, 0.016, 0.019 and 0.024 for 80, 130, 180 and 230 μA, respectively, Figure 2C).

The contralateral septal and intermediate HC became activated only at higher amplitudes (Figure 2A). However, the fMRI time courses of these areas illustrate that there was already a weak response at 80 μA and only minor changes occurred at higher amplitudes (Figure 2D).

Overall, the induced activity patterns were very similar across all current amplitudes.

### 3.3 Effect of the stimulation frequency on fMRI activation in epileptic mice

**Figure 3:**
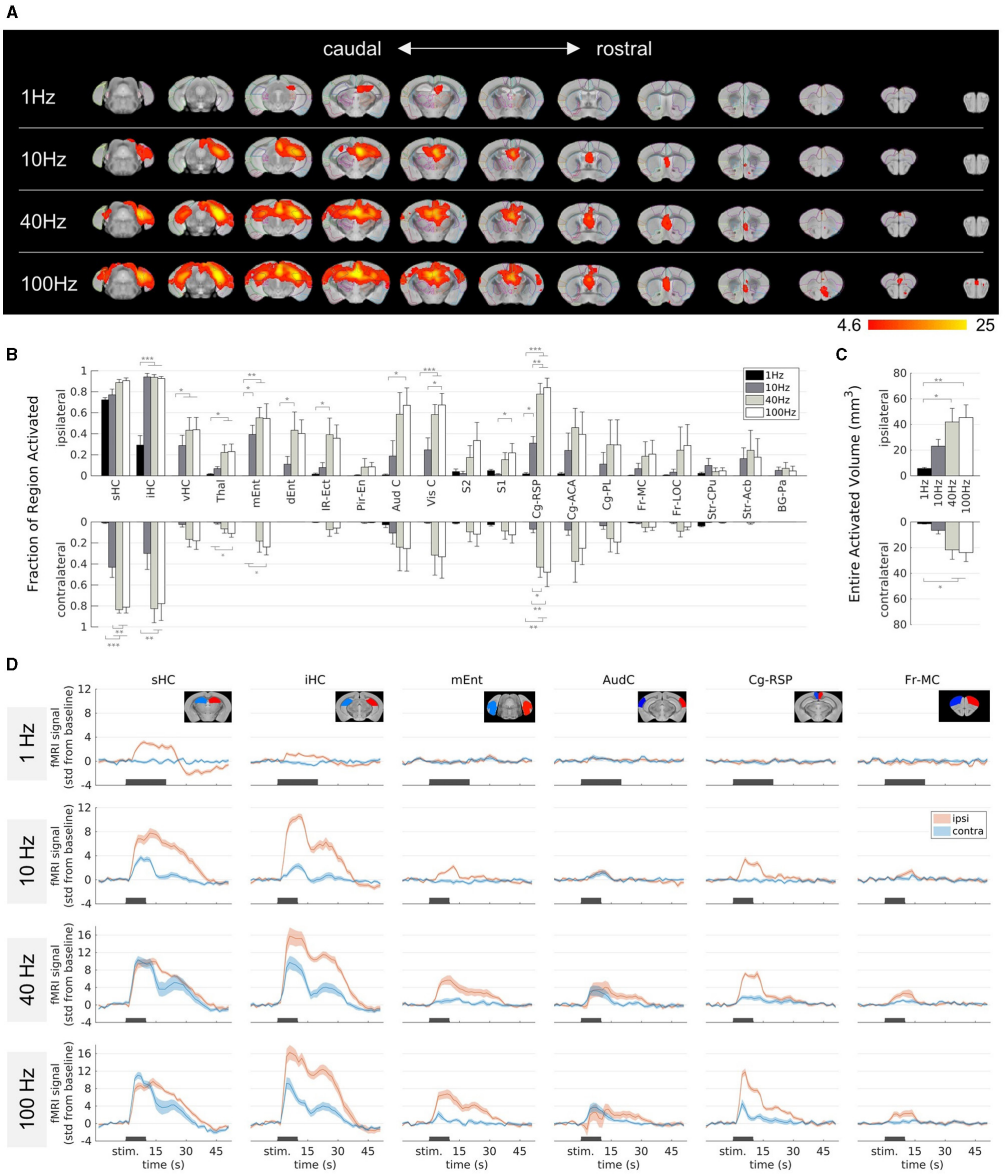
Effect of the stimulation frequency on fMRI activation in epileptic mice. **(A)** fMRI activation maps for stimulation frequencies of 1 Hz (top row) to 100 Hz (bottom row) from caudal **(left)** to rostral **(right)** direction. The group-level fMRI activations are shown as red-yellow overlays in the AMBMC reference space (gray background) with contours of the AMBMC mouse brain atlas (colors red to yellow represent z-scores from 4.6 to 25 of significant responses at a voxel-wise corrected threshold *p* < 0.05; group *n* = 6 for 1 and 10 Hz, *n* = 4 for 40 and 100 Hz). **(B)** Quantification of the activated fractions in atlas regions showed with increasing frequency several significant changes (*p < 0.05, **p < 0.01, ***p < 0.001, one-way ANOVA with Tukey-Kramer multiple comparison tests). **(C)** Quantification of the entire activated volume on the ipsilateral and contralateral hemisphere showed significant increases from 1–10 to 40–100 Hz (*p < 0.05, **p < 0.01, one-way ANOVA with Tukey-Kramer multiple comparison tests). The activated volumes differed significantly between the ipsi- and contralateral hemispheres at frequencies of 1 and 10 Hz, but not at 40 and 100 Hz (one-sample *t*-tests between ipsi- and contralateral “Entire Activated Volumes”; Bonferroni-Holm corrected *p* = 0.03, 0.016, 0.069 and 0.076 for 1, 10, 40, and 100 Hz, respectively. **(D)** Mean BOLD fMRI responses in selected regions (mean ± SEM). The black bars indicate the stimulation periods.

Next, we investigated the impact of different stimulation frequencies in chronically epileptic mice. We kept the amplitude constant at 130 μA and stimulated at 1, 40, and 100 Hz (Figure 3).

At 1 Hz, activation was visible only around the stimulation site, whereas with increasing frequency, activation spread from the HC to large parts of the brain (Figure 3A). At 100 Hz, fMRI responses were detected in the thalamus, several cortical, cingulate and frontal regions. The activated fractions in several brain regions (Figure 3B) and the total activated volumes increased significantly with frequency (Figure 3C, one-way ANOVA *p* < 0.05 with Tukey-Kramer multiple comparison tests).

Another noteworthy change from 10 Hz to 40–100 Hz was the stronger recruitment of the contralateral hemisphere, for example in the intermediate HC, the medial entorhinal cortex, and the cingulate areas. The activated volumes differed significantly between the ipsi- and contralateral hemispheres at frequencies of 1 and 10 Hz, but not anymore at 40 and 100 Hz (one-sample *t*-tests between ipsi- and contralateral “Entire Activated Volumes”; Bonferroni-Holm corrected *p* = 0.03, 0.016, 0.069, and 0.076 for 1, 10, 40, and 100 Hz, respectively, Figure 3C).

The fMRI time courses show that the response at 1 Hz was comparatively weak (Figure 3D). The longer stimulation duration (20 s) was chosen because it was unclear how weak the response would be, but Figure 3D shows that the BOLD response is clearly detectable already after 10 s.

The evaluated fMRI patterns showed only little inter-animal variability (Supplementary Figure 5). In one epileptic mouse, a 40 Hz stimulation elicited seizures on subsequent days (Supplementary Figure 6). In contrast to the ADs in control mice, the seizures in the epileptic mouse were associated with strong seizure-induced movements. However, the rarity of these seizures in only one subject precluded a systematic analysis.

Overall, the stimulation frequency decisively determined which brain regions were affected by the stimulation. While the effect of 1 Hz stimulation was mainly limited to the stimulation site, increasing the frequency to 100 Hz led to a spread across the whole brain, including the contralateral HC, cortical and frontal regions.

### 3.4 Changes in fMRI activation due to prolonged 1 Hz stimulation

**Figure 4:**
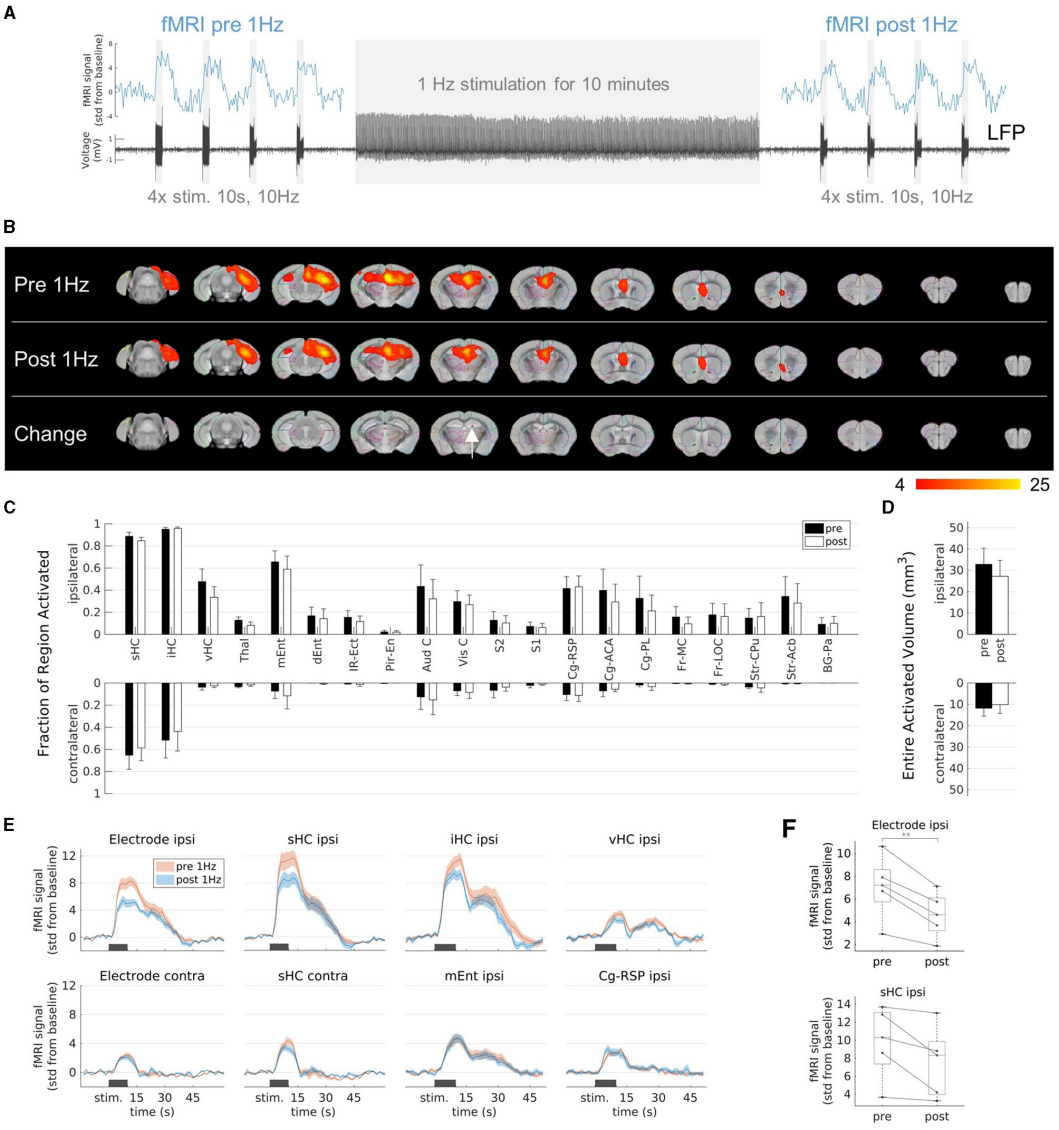
Effects on fMRI response to prolonged 1 Hz stimulation. **(A)** Experimental design. Stimulation at 1 Hz (130 μA) was applied for 10 min and block-design fMRI experiments (4 stimulation blocks for 10 s at 10 Hz and 130 μA) were performed before and after the 1 Hz stimulation to evaluate the activation patterns. **(B)** fMRI activation maps before (first row, “Pre 1 Hz”), after the 1 Hz stimulation (second row, “Post 1 Hz”), and the group-level comparison between them (third row). A significant decrease (two-sample paired *t*-test, voxel-wise corrected threshold *p* < 0.05; group *n* = 5) was detected in a small area (red color) within the ipsilateral septal HC (white arrow). **(C)** Quantification of the activated fractions in atlas regions (mean ± SEM). There were no significant changes from pre 1 Hz to post 1 Hz (paired *t*-tests *p* > 0.05). **(D)** Quantification of the entire activated volumes on the ipsilateral and contralateral hemisphere (mean ± SEM). There was no significant change from pre 1 Hz to post 1 Hz (paired *t*-tests *p* > 0.05). **(E)** Average fMRI responses in selected regions (mean ± SEM). The fMRI response after the 1 Hz stimulation (blue) was reduced compared to the fMRI response before the 1 Hz stimulation (red) in the regions-of-interest (ROIs) near the stimulation electrode (ROIs “Electrode ipsi” and “sHC ipsi”), whereas no change was observed in ROIs far from the stimulation site (e.g., at the contralateral side or in the entorhinal cortex and cingulate region). The ROIs “Electrode ipsi” and “Electrode contra” correspond to a volume of 0.5 mm^3^ (eight voxels) directly around the electrodes. These and the ROIs in the septal HC (“sHC ipsi” and “sHC contra”) were manually drawn in the single subject space to exclude the electrode artifacts. **(F)** Change of fMRI amplitudes in the ROIs “Electrode ipsi” (paired *t*-test between pre and post 1 Hz: **p < 0.01) and “sHC ipsi” (paired *t*-test: *p* 0.06). Mean fMRI signals in a 10 s window shifted by 5 s from the start of the stimulation to account for the delayed fMRI response.

We found that 1 Hz stimulation induced local fMRI activity. However, prolonged application, such as in Paschen et al. (2023), may additionally influence excitability or connectivity. Both would be detectable by modified fMRI patterns. We therefore compared the fMRI patterns induced by 10 Hz block-design fMRI before and after prolonged 1 Hz stimulation (Figure 4A).

The extent of the fMRI pattern before 1 Hz stimulation was similar to that after 1 Hz stimulation (Figure 4B). However, the group comparison revealed a significant decrease within the ipsilateral septal HC (Figure 4B, third row). There were no significant changes in the activated fraction in individual regions (Figure 4C) or in the total volumes (Figure 4D).

To further clarify this finding, we compared the fMRI time courses before and after the 1 Hz stimulation in volumes directly around the electrode, in the entire septal, intermediate and ventral HC (Figure 4E). We found that the fMRI response in the areas close to the stimulation electrode were reduced after the 1 Hz stimulation and this effect became weaker with increasing volume and distance from the electrode. More distant regions, such as the entorhinal cortex, the cingulate region or the contralateral HC showed no change. The fMRI response was not only reduced on average, but also in each animal (paired *t*-tests; p = 0.004 in volume “Electrode ipsi”, p = 0.06 in volume “sHC ipsi”, Figure 4F).

Regions that were not affected by the 1 Hz stimulation but only by the 10 Hz stimulation served here as negative control. A change in physiological parameters (e.g., depth of anesthesia, heart rate) or simply the time could theoretically have altered the BOLD fMRI response from before to after the 1 Hz stimulation. However, there are several regions, which were not affected by the 1 Hz stimulation, for example mEnt, Cg-RSP or the contralateral septal HC near the electrode (see Figure 3D, Supplementary Figure 5). These regions responded only to the 10 Hz stimulations and showed unchanged BOLD responses after the 10 min 1 Hz stimulation (Figure 4E). From this, it can be concluded that the 1 Hz stimulation was mainly responsible for the local reduction of the fMRI response.

## 4 Discussion

This study systematically investigated brain-wide fMRI activation patterns under various stimulation protocols in both chronically epileptic and sham control mice. Importantly, our results were derived from the ihKA mouse model of mTLE that replicates several key features of the human disease, such as hippocampal sclerosis and recurrent seizures (Bouilleret et al., 1999).

To compare different stimulation frequencies, *in vivo* studies must necessarily be limited to a few frequencies to be tested due to time constraints. We chose the intermediate frequencies of 10/40 Hz because there are numerous studies for this frequency range, but they were performed in healthy animals (Moreno et al., 2016) or stimulated different structures such as the perforant path (e.g. Angenstein et al., 2007; Canals et al., 2008; Riemann et al., 2017). In conventional DBS applications in epilepsy, usually higher frequencies ≥100 Hz are applied (Zangiabadi et al., 2019). However, the benefit of low frequency stimulation in epilepsy is also currently being investigated (Paschen et al., 2020, 2023; Manzouri et al., 2021). Therefore, in this study we also included a high (100 Hz) and a low (1 Hz) frequency for comparison.

Other studies have investigated the frequency dependent propagation of activity induced in the HC either via DBS (Moreno et al., 2016) or optogenetics (Weitz et al., 2015). Interestingly, these studies in healthy animals show an increased spread from the HC to extrahippocampal areas only in the frequency range 5 Hz to around 40 Hz and a plateau or even blockade at 40 Hz and higher frequencies. In contrast, our results from the ihKA mouse model of mTLE showed at 100 Hz stimulation a wide spread from the dorsal HC to several cortical areas. MTLE with unilateral hippocampal sclerosis is associated with hyperexcitability, cell loss and rewiring (Häussler et al., 2012; Marx et al., 2013; Janz et al., 2017a), which may explain the wider spread of activity. This is also consistent with increased functional connectivity that has been found in other rodent models of mTLE by resting-state fMRI (Gill et al., 2017; Li et al., 2021).

Aiming to interfere with epileptiform activity, recent studies explored the application of low frequency stimulation in both human mTLE (Koubeissi et al., 2013, 2022; Lim et al., 2016) and rodent mTLE models (Rashid et al., 2012; Paschen et al., 2020, 2023). A possible explanation for the benefit in focal epilepsy is that prolonged stimulation of the epileptic focus leads to long-term depression and reduced excitability (Durand and Bikson, 2001; Manzouri et al., 2021), which may be associated with reduced seizure incidence. Our recent study on the ihKA mouse model of mTLE has also shown that 1 Hz stimulation can reduce seizures without affecting memory or behavioral performance (Paschen et al., 2023). In the current study, we showed using fMRI, that 1 Hz stimulation has a local effect, which may explain the minimal side effects, and yet is able to reduce local excitability, supporting this strategy to interfere with epileptiform activity. The relatively weak effect on fMRI response in this study was most likely due to the relatively short application time of 10 minutes, which can be extended in the future to further investigate if changes in fMRI response increase.

A very pronounced difference to the sham controls in our study was that the induced activity in epileptic animals was extremely strong in the ipsilateral HC and substantially weaker on the contralateral side. This is consistent with known morphological changes in the ihKA mouse model. Hippocampal sclerosis is characterized by cell loss e.g. in CA1, CA3 and the hilus of the septal ipsilateral HC (Lévesque and Avoli, 2013; Marx et al., 2013). However, the DG mainly shows a loss of inhibitory interneurons. Excitatory granule cells in the DG are spared and undergo structural reorganization, a process known as mossy fiber sprouting (Bouilleret et al., 1999). A recent study using acute slices in the ihKA mouse model has indeed shown increased excitability of the DG due to increased synaptic strength, the formation of recurrent synapses, and a reduction of inhibitory synapses on the granule cells (Vergaelen et al., 2024). This finding may have a direct influence on the assessment of stimulation results. The efficacy of low frequency stimulation was found to vary from patient to patient (Lim et al., 2016) or not to be beneficial (Boëx et al., 2007). Reasons why such a local stimulation may be ineffective could be that the seizure focus is not directly at the electrode or that there are additional foci, e.g. in bilateral TLE (Aghakhani et al., 2014; Chiang et al., 2022). Approaches to improve efficacy were to target both hippocampi either by the stimulation of a fiber tract connected to bilateral hippocampi (Koubeissi et al., 2022) or by bilateral stimulation via several electrodes (Cukiert et al., 2014, 2021). Even in unilateral TLE, switching from unilateral to bilateral DBS could improve the efficacy in some patients (Vonck et al., 2013). For the ihKA model in particular, a recent study provides evidence that the contralateral HC plays an important role in the generation of seizures (Padmasola et al., 2024). The fMRI results in sham controls would suggest that an ipsilateral stimulation already affects the contralateral HC. However, the results in epileptic animals showed that the BOLD activity induced by stimulation, especially at low frequency, does not spread equally to the contralateral side, explaining the benefit of bilateral stimulation.

The stimulation parameters can also be adjusted to improve the effect of the stimulation. The results of the present study show that the stimulation frequency constitutes the key factor in determining which areas/networks of the brain are affected by a stimulation. The extent of activation could be adjusted from local activation at 1 Hz to brain-wide activation at 100 Hz. In contrast, we found that the stimulation amplitude had little influence on the extent of the induced fMRI patterns over a relatively wide amplitude range. 80 μA was the lowest amplitude in our study that evoked clearly detectable fMRI responses in individuals and higher currents led only to a minor, yet unspecific broadening of the activation pattern, but did not recruit fundamentally new areas. This is consistent with a previous clinical study that compared two current levels (4 and 8 mA) and found no difference in activation patterns (Jones et al., 2014). The absolute values of the stimulation current may differ between studies due to differences in electrode design and/or positions, but it can be concluded that if a response to a stimulation is detectable, modifying the current strength is unlikely to result in a significantly different response or functional outcome. One point to note here, however, is the low spatial specificity of the electrical stimulation in this study. Already with 80 μA stimulation, the induced fMRI activation extended over the entire septal ipsilateral HC. On the one hand, it would be possible to investigate more precisely at what point stimulation becomes visible, whereby the exact threshold probably varies from individual to individual and also depends on the frequency. On the other hand, newer electrode designs, so-called directional DBS electrodes (Steigerwald et al., 2019), could be used to control the current flow more precisely. For example, with control in a more horizontal direction, one could aim to stimulate only individual HC subfields. The sclerotic HC in particular shows subfield-specific changes, e.g. loss of pyramidal neurons in CA1/CA3 regions and preservation of dentate granule cells with their entorhinal inputs, which is why this spatially more precise electrical stimulation would be interesting to investigate.

At the beginning of the study, we assumed that stimulation above a threshold, a certain amplitude or frequency, would reliably trigger epileptic seizures. Therefore, we chose the stimulation design from low to high amplitudes/frequencies (as opposed to a randomized design). To minimize bias from one experiment to the next, we applied only four blocks of stimulation in each experiment. One surprising result of our study was that the stimulations triggered epileptiform ADs in sham controls, whereas in epileptic animals, seizures occurred in only one animal. This represented only 4.1 % (2 of 49) of all stimulation experiments in epileptic mice and was therefore not representative. It is still unknown how exactly epileptic seizures are triggered. The seizures themselves, in epileptic animals as well as patients, are difficult to study with fMRI because they occur spontaneously and are often associated with strong movements. The fact that the stimulations elicited epileptiform ADs only in sham controls and that these animals did not move during the ADs, suggests that the mechanisms of ADs in sham controls and spontaneous epileptic seizures are different. The results from studies of stimulated ADs in healthy animals, e.g. (Airaksinen et al., 2010, 2012; Duffy et al., 2015, 2020), have to be translated to real epilepsy with caution. However, the comparison to healthy animals can of course provide valuable information about structural alterations. In this study, the comparison was limited because ADs in controls occurred already at 10 Hz. For a direct comparison, also at higher frequencies, it is therefore necessary to select stimulation lengths in both epileptic and control animals that are sufficiently short ( ≤ 1s) to avoid causing seizures.

BOLD fMRI was acquired with a gradient multi-echo EPI sequence in this study. The multi-echo approach should provide optimal sensitivity to T2^*^, but the temporal resolution (TR 1.5 s) could be improved in future studies. Chuang et al. developed a multiband EPI sequence for ultrafast fMRI (TR 300 ms) for preclinical scanners (Lee et al., 2019; Chuang et al., 2023). However, in BOLD fMRI, which does not directly measure neuronal activity, the limiting factor is the hemodynamic coupling, which leads to delayed and sluggish responses in relation to the actual neuronal activity. Recently, the possibility of directly detecting neuronal activity using fMRI has been reported (Toi et al., 2022). This method is not yet sufficiently established (Choi et al., 2024), but could possibly provide more insights into activation and propagation of activity in stimulation studies.

One limitation of this study is that we only examined which regions respond to stimulation and not to what extent the stimulation patterns are related to the efficacy of the stimulation in suppressing epileptic activity. A future study could shed more light on the relation between stimulated patterns, reduction of epileptic activity, occurrence of adverse effects and the possibility to adapt the stimulation parameters in individuals to tailor the outcome. The positive benefit of DBS in epilepsy could possibly occur only after some time and a distinction would have to be made between acute and chronic effects. Therefore, a long-term study with DBS applications e.g. once a day or constantly every few minutes would be optimal. With long-term EEG to quantify the epileptic activity and fMRI at several time points, the changes over time can be determined. To detect possible adverse effects of stimulation, behavioral tests, such as in (Paschen et al., 2023), should be included. This would be the best way to correlate the induced activity with beneficial effects (reduction of epileptic activity) and adverse effects (worse scores in the behavioral test). Our hypothesis is that this would allow us to identify which stimulation patterns lead eventually to optimal results.

Another limitation is that this study was performed only in an animal model of mTLE. In the human disease, there is greater variability between patients, the epileptic foci might not be so restricted and there is no guarantee that the electrode is positioned exactly at the focus. This variability underscores the benefit of controlling stimulation responses using fMRI, which may provide a way to more effective, individualized treatment.

An extension of this study would be to investigate other forms of stimulation, e.g. DC stimulation which is also used in epilepsy (Fregni et al., 2006; Sudbrack-Oliveira et al., 2021) or very high stimulation frequencies in the kHz range (Neudorfer et al., 2021). A very interesting recent development is the so-called temporal interference (TI) stimulation (Grossman et al., 2017), which makes it possible to stimulate non-invasively, yet focally and even deep enough to reach the human HC (Violante et al., 2023). Applications in the mouse model of epilepsy have also shown that TI stimulation can be effective enough to reduce epileptic activity (Acerbo et al., 2022, 2024). It remains to be investigated whether non-invasive TI stimulation can produce similar effects as stimulation via implanted electrodes.

## 5 Conclusions

In this study, we presented the brain-wide responses to hippocampal stimulations in the ihKA model of mTLE. When estimating the potential outcome of a stimulation in mTLE, it is essential to consider changes in the epileptic brain and the impact of stimulation parameters. Compared to sham controls, epileptic mice showed pronounced responses in the ipsilateral HC and a wider spread to the entorhinal cortex and septal region. The key parameter that determined the extent of activation was the stimulation frequency. While low frequency (1 Hz) stimulation elicited only a local fMRI response, high frequency (40–100 Hz) stimulation resulted in spread of activity from the HC to cortical regions in the epileptic brain. The amplitude parameter, in contrast to frequency, offered little opportunity to vary the stimulation effects. However, the fact that the amplitude did not show a large influence makes it possible to compare the results of studies even if the amplitude settings differ. This supports the translational value of preclinical fMRI studies.

## Data availability statement

The datasets presented in this study can be found in online repositories. The names of the repository/repositories and accession number(s) can be found below: https://doi.org/10.18112/openneuro.ds004959.v1.0.0.

## Ethics statement

The animal study was approved by Regierungspräsidium Freiburg, Germany. The study was conducted in accordance with the local legislation and institutional requirements.

## Author contributions

NS: Conceptualization, Data curation, Formal analysis, Investigation, Methodology, Software, Visualization, Writing – original draft, Writing – review & editing. EP: Conceptualization, Data curation, Investigation, Methodology, Writing – review & editing. PL: Funding acquisition, Writing – review & editing. DE: Funding acquisition, Project administration, Resources, Writing – review & editing. CH: Funding acquisition, Project administration, Resources, Supervision, Writing – review & editing.
