# Supplementary material for: Probing hippocampal stimulation in experimental temporal lobe epilepsy with functional MRI
Source: Front Neuroimaging. 2024 Aug 14;3:1423770. doi: 10.3389/fnimg.2024.1423770 (PMC11349577; doi:10.3389/fnimg.2024.1423770)
Supplement: Supplementary file 1 [file Data_Sheet_1.docx]

Supplementary material

**Supplementary table 1: Overview of the sessions/tests performed on each animal**. “y”: scan successful, “x”: scan aborted, “-“ not started, “AD”: epilepiform afterdischarges (AD) recognized.

| Session: “Amplitude”, kainate mice | | | | | |
| --- | --- | --- | --- | --- | --- |
| **Animal ID** | **30 µA** | **80 µA** | **130 µA** | **180 µA** | **230 µA** |
| KA-1 | y | y | y | x | - |
| KA-2 | - | y | y | y | y |
| KA-3 | - | y | y | x | - |
| KA-4 | - | y | y | y | y |
| KA-5 | - | y | y | y | y |
| KA-6 | - | y | y | y | y |
| Session: “Amplitude”, control mice | | | | | |
| **Animal ID** | **30 µA** | **80 µA** | **80 µA** |  |  |
| CO-1 | - | AD | y |  |  |
| CO-2 | y | AD | y |  |  |
| CO-3 | y | AD | y |  |  |
| CO-4 | y | AD | y |  |  |
| Session: “Frequency”, kainate mice | | | | | |
| **Animal ID** | **1 Hz** | **40 Hz** | **100 Hz** |  |  |
| KA-1 | y | y | y |  |  |
| KA-2 | y | y | y |  |  |
| KA-3 | y | AD | - |  |  |
| KA-5 | y | y | y |  |  |
| KA-6 | y | y | y |  |  |
| Session: “pre-post 1Hz”, kainate mice | | | | | |
| **Animal ID** | **10 Hz** | **1 Hz** | **10 Hz** |  |  |
| KA-1 | y | y | y |  |  |
| KA-2 | y | y | y |  |  |
| KA-3 | y | y | y |  |  |
| KA-5 | y | y | y |  |  |
| KA-6 | y | y | y |  |  |

**
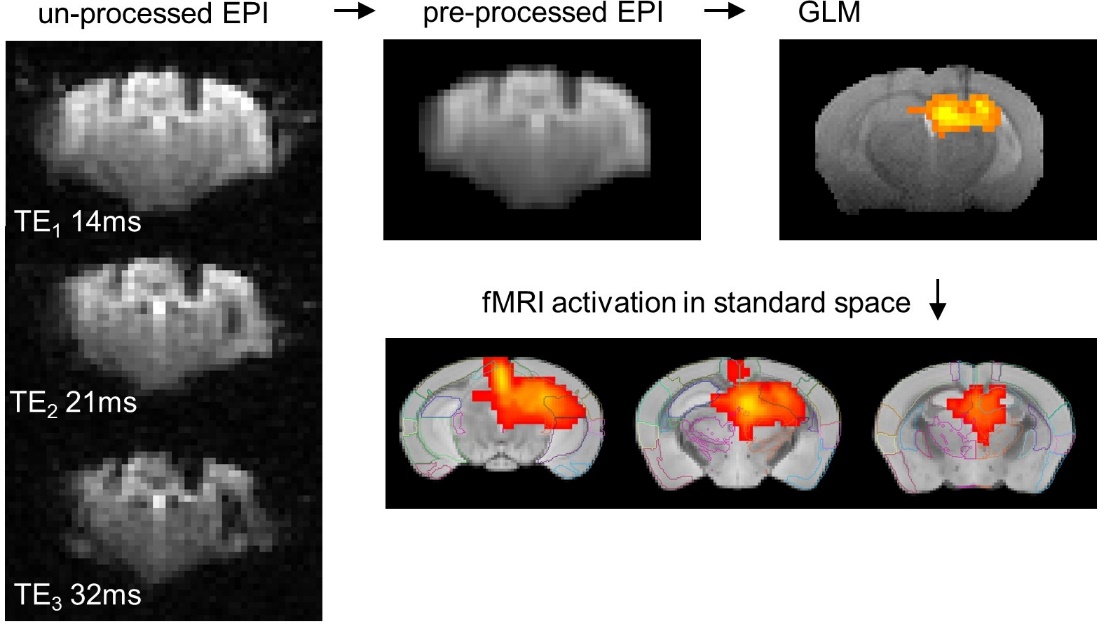
Supplementary figure 1: Basic post-processing workflow.** The three unprocessed echo images of one slice are shown on the left, also revealing the electrode artifacts. Preprocessing (motion and slice timing correction, echo combination, temporal and spatial filtering) generated a 4D data set for further analysis. General linear modeling yielded the fMRI activations registered into standard space. See methods section for details.


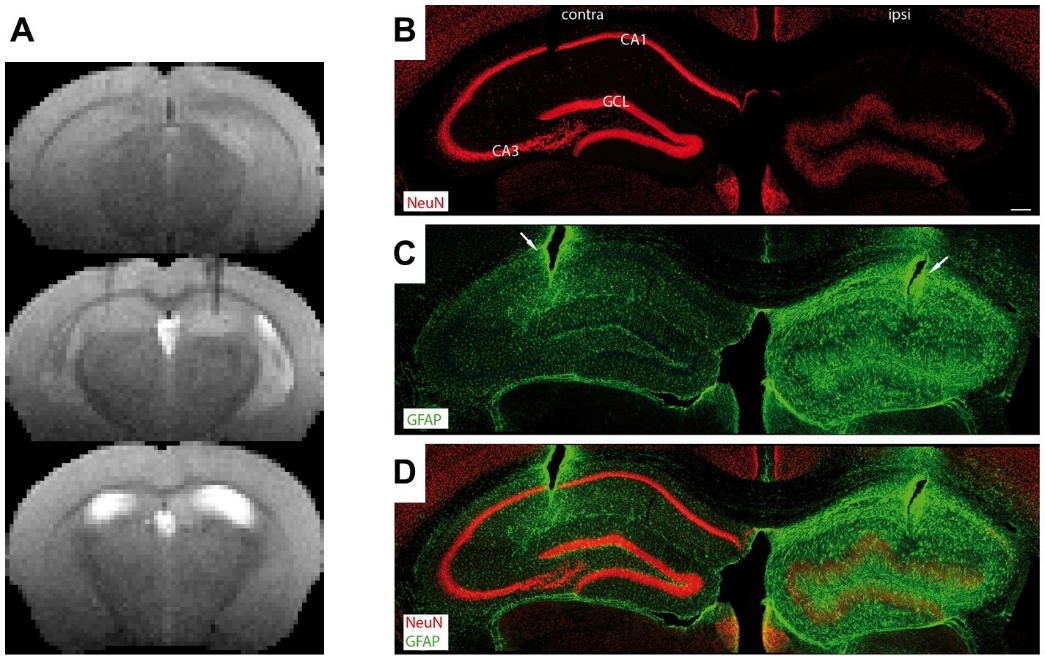


**Supplementary figure 2: Representative in-vivo MRI images and immunohistochemistry. (A)** Three adjacent slices (top to bottom) of the T2-weighted imaging (RARE sequence) showing the electrode artefacts in the middle slice. **(B-D)** Representative section of the contra-and ipsilateral hippocampus double immunolabeled for NeuN/ GFAP at 30 days following kainate injection. **(B)** Immunolabeling for NeuN which visualizes neuronal cell bodies. Note the neuronal loss in CA1, CA3 and hilus and the dispersion of the granule cell layer (GCL), all characteristic features of hippocampal sclerosis. **(C)** Immunolabeling for GFAP which shows astrocyte activation and gliosis. Note the strong activation of astrocytes along the electrode tracks (arrows) in both hippocampi and the strong gliosis in the ipsilateral hippocampus in regions of cell death and the dispersed GCL. **(D)** Merged pictures of NeuN and GFAP immunolabeling. Scale bar: 200 µm; CA, cornu ammonis; GCL, granule cell layer; GFAP, glial fibrillary acidic protein; NeuN, neuronal nuclei.


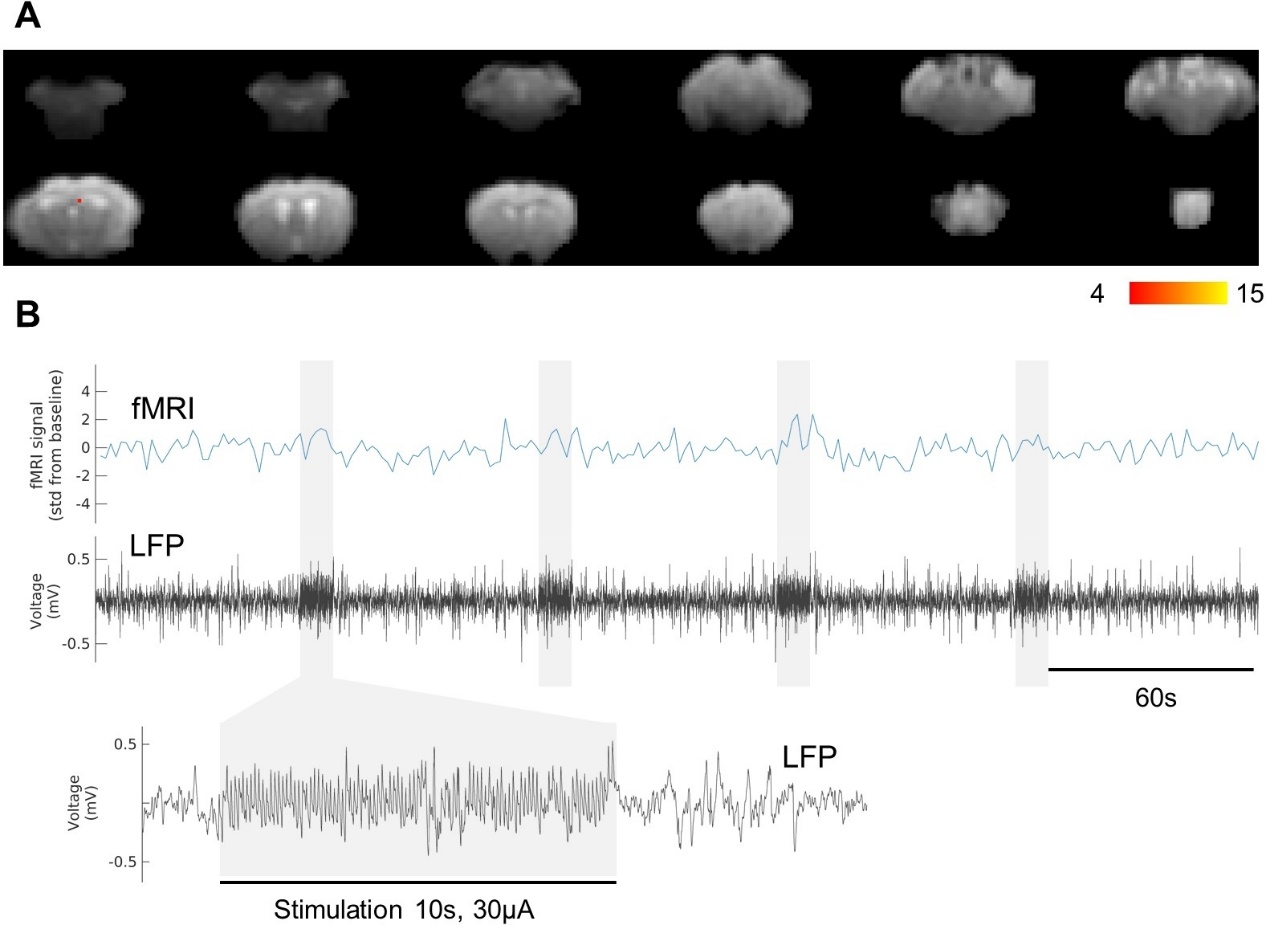


**Supplementary figure 3: Stimulation in a kainate mouse with 30 µA at 10 Hz. (A)** fMRI activation (red-yellow overlay, voxel-wise corrected threshold p <0.05) in an epileptic mouse from caudal (top left) to rostral (bottom right) direction at stimulation parameters: 10 Hz, 10 s, 30 µA. (**B)** While the stimulation artifacts in the LFP (black trace) confirm the applied stimulation, the responses in the fMRI (blue, trace of the single activated voxel shown in A) are only minimal at this current amplitude.


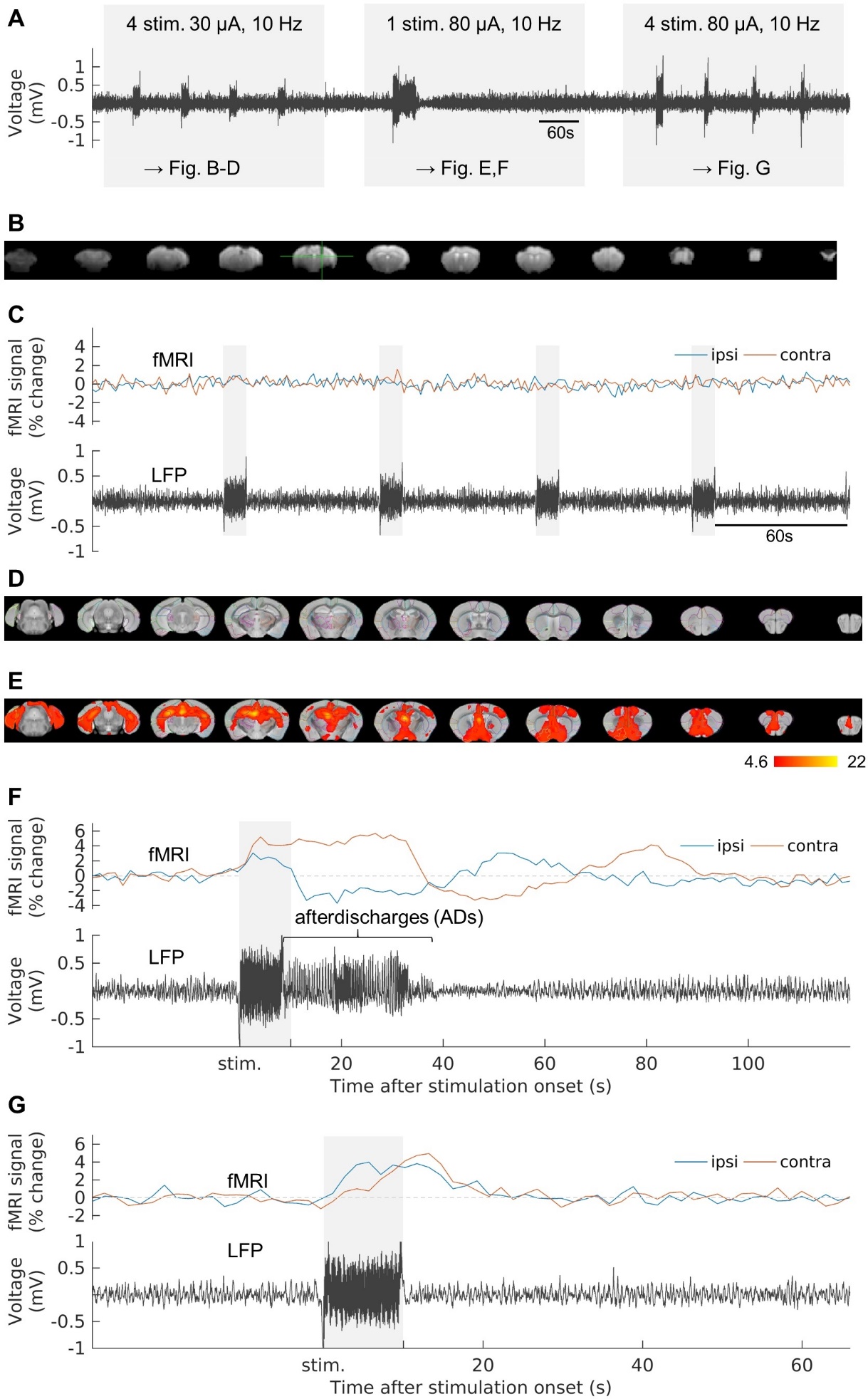


**Supplementary figure 4: Stimulations in sham controls. (A)** Experiments in sham controls consisted of a first block-design experiment with stimulations at 30 µA, followed by two stimulation experiments at 80 µA. (**B)** single-level GLM analysis resulted in no activation at 30 µA (slices from caudal (top left) to rostral (bottom right), representative control). (**C)** While the stimulation artifacts in the LFP (black trace) confirm the applied stimulations at 30 µA, no fMRI responses were detected (blue and orange traces from the ipsi- and contralateral septal HC, respectively). (**D)** Group-level (n=3) analysis also showed no activation at 30 µA. (**E, F)** The first stimulation at 80 µA triggered afterdischarges (ADs) in sham controls. This was detected in the LFP recordings during the fMRI scanning and the remaining three stimulations were omitted. (**E)** Group-level (n=4) fMRI response of the ADs in control animals (colors red to yellow represent z-scores of significant responses at a voxel-wise corrected threshold p <0.05). (**F)** FMRI (blue and orange traces from the ipsi- and contralateral septal HC, respectively) and LFP trace of a representative AD in a control mouse. (**G)** The block design experiments following the ADs did not trigger further ADs, which was confirmed by the LFP (black trace). These block-design experiments were used for GLM analysis (see Figure 1C).


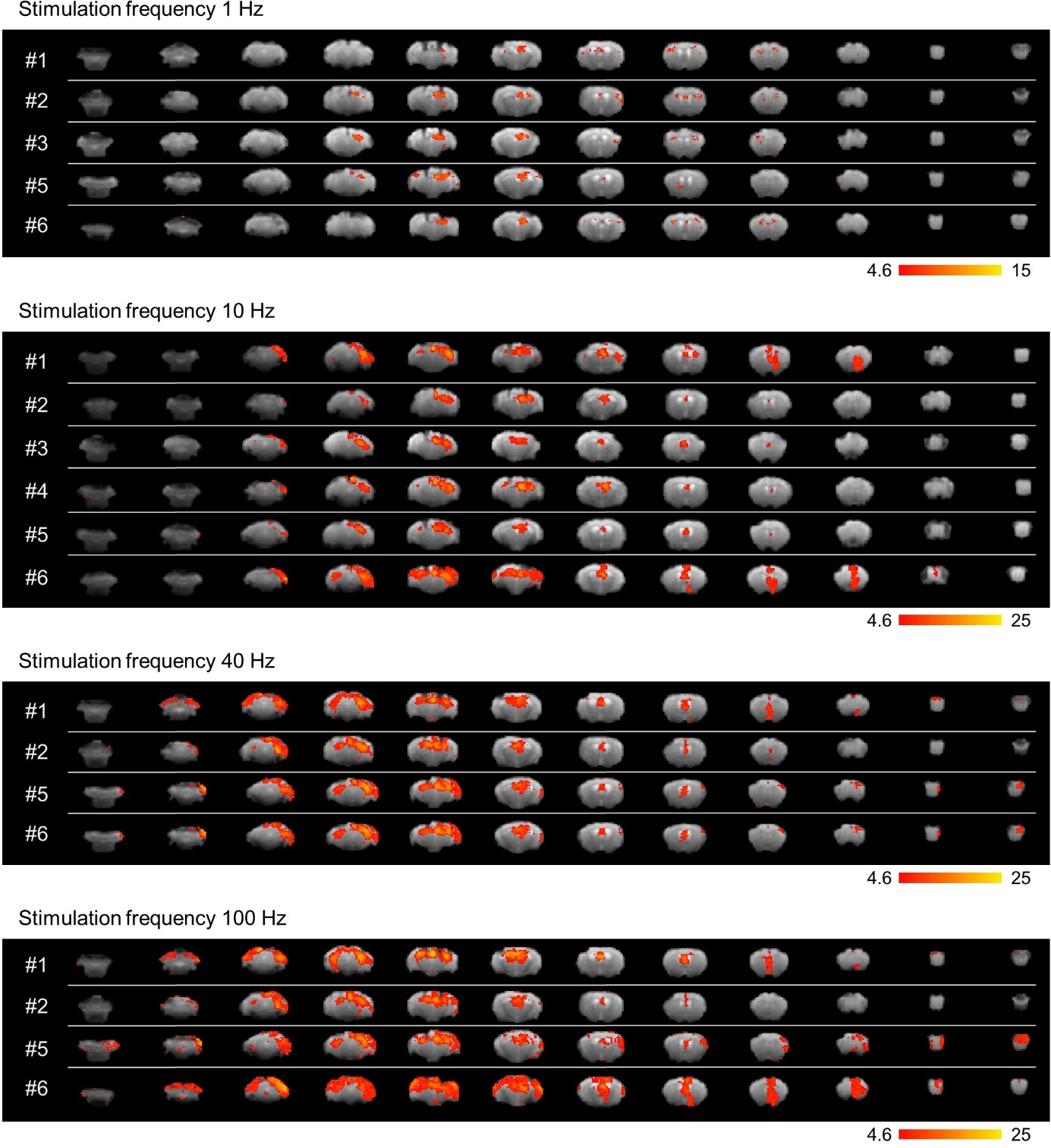
**Supplementary figure 5:** **Individual fMRI activation maps of kainate mice.** FMRI activations (at 130 µA, 1-100 Hz) of kainate mice overlaid on an fMRI volume of this experiment (colors red to yellow represent z-scores of significant responses at a voxel-wise corrected threshold p <0.05). One mouse (#4) died during the period of the experiments (but not during or closely after an fMRI measurement), and one mouse (#3) exhibited epileptic seizures at 40 Hz and was therefore not included in the group analysis of 40 and 100 Hz.


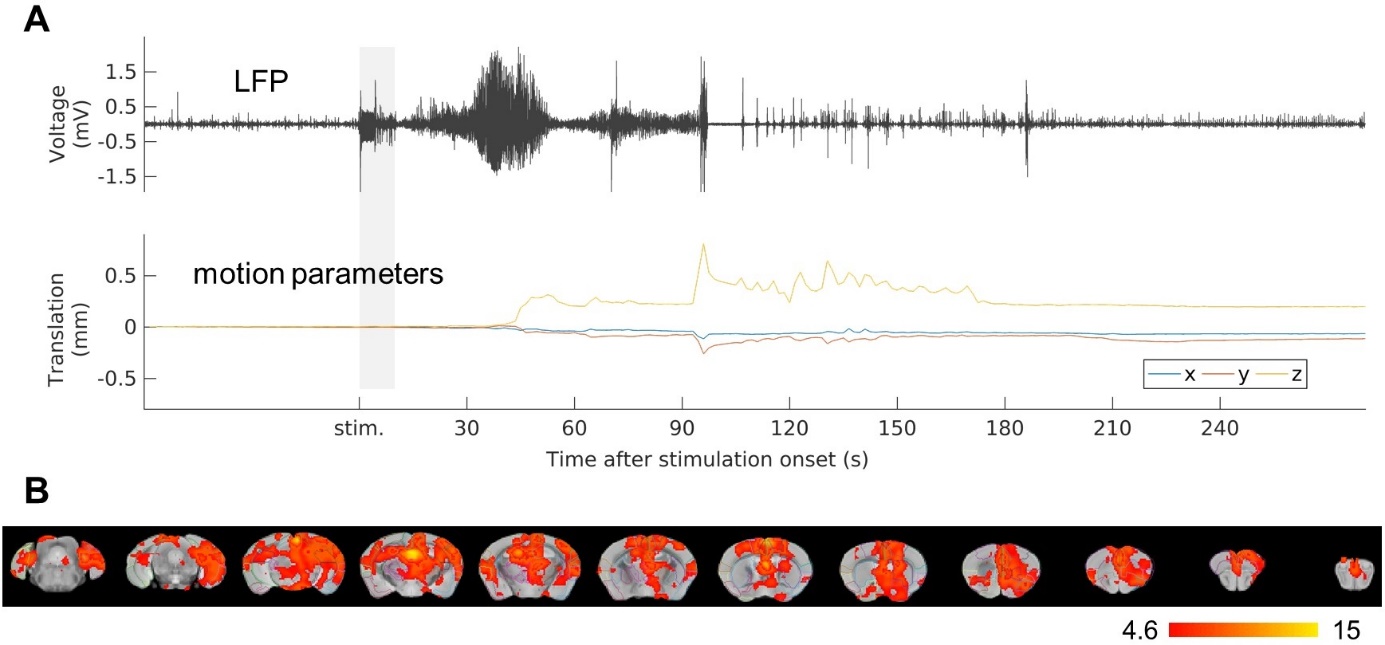
**Supplementary figure 6: Stimulations at 40 Hz elicited seizures in one kainate mouse. (A)** The LFP trace showed epileptiform activity triggered by the electrical stimulation (parameters 40 Hz, 10s, 130 µA). Approx. 30 s after seizure onset, strong seizure-induced movements occurred (visible in the motion parameters estimated by the FSL function “mcflirt”). (**B)** FMRI analysis of the first 30 s of the two seizures (colors red to yellow represent z-scores of significant responses at a voxel-wise corrected threshold p <0.05).
